# chiLife: An open-source Python package for *in silico* spin labeling and integrative protein modeling

**DOI:** 10.1371/journal.pcbi.1010834

**Published:** 2023-03-31

**Authors:** Maxx H. Tessmer, Stefan Stoll

**Affiliations:** Department of Chemistry, University of Washington, Seattle, Washington United States of America; University of Maryland School of Pharmacy, UNITED STATES

## Abstract

Here we introduce chiLife, a Python package for site-directed spin label (SDSL) modeling for electron paramagnetic resonance (EPR) spectroscopy, in particular double electron–electron resonance (DEER). It is based on *in silico* attachment of rotamer ensemble representations of spin labels to protein structures. chiLife enables the development of custom protein analysis and modeling pipelines using SDSL EPR experimental data. It allows the user to add custom spin labels, scoring functions and spin label modeling methods. chiLife is designed with integration into third-party software in mind, to take advantage of the diverse and rapidly expanding set of molecular modeling tools available with a Python interface. This article describes the main design principles of chiLife and presents a series of examples.

## Author summary

Thanks to modern modeling methods like AlphaFold2, RosettaFold, and ESMFold, high-resolution structural models of proteins are widely available. While these models offer insight into the structure and function of biomedically and technologically significant proteins, most of them are not experimentally validated. Furthermore, many proteins exhibit functionally important conformational flexibility that is not captured by these models. Site-directed spin labeling (SDSL) electron paramagnetic resonance (EPR) spectroscopy is a powerful tool for probing protein structure and conformational heterogeneity, making it ideal for validating, refining, and expanding protein models. To extract quantitative protein backbone information from experimental SDSL EPR data, accurate modeling methods are needed. For this purpose, we introduce chiLife, an open-source Python package for SDSL modeling designed to be extensible and integrable with other Python-based protein modeling packages. With chiLife, appropriate SDSL EPR experiments for protein model validation can be designed, and protein models can be refined using experimental SDSL EPR data as constraints.

This is a *PLOS Computational Biology* Software paper.

## 1. Introduction

Site-directed spin labeling (SDSL) electron paramagnetic resonance (EPR) is a powerful method for investigating protein structure and dynamics [1–3]. Solution continuous-wave (CW) EPR probes global motions, like protein tumbling, and local motions, like side chain and backbone dynamics [4,5], which provide valuable information on protein topology, tertiary and quaternary structure, and functionally important protein dynamics. Power saturation EPR measures spin label solvent accessibility and membrane depth [6,7], which can provide information on protein transmembrane insertion and topology and inform protein–membrane docking of peripheral membrane proteins [8–10]. Pulse dipolar EPR experiments such as double electron–electron resonance (DEER) determine distance distributions between pairs of spin labels [11–15]. These distance distributions directly provide information on protein tertiary and quaternary structure. Coupled with high-resolution structural techniques, SDSL EPR provides insight into protein conformational landscapes and how they change in response to different stimuli [16–27]. Accordingly, SDSL EPR is particularly useful for validation and refinement of protein structural models as well as expansion of these models to include conformational heterogeneity and distinct alternate conformational states. SDSL EPR has been used to validate, refine, and expand upon models developed using experimental structure determination methods such as x-ray crystallography and cryo-electron microscopy. Now, with modern computational methods like AlphaFold2, RosettaFold and ESMFold [28–31], SDSL EPR have become even more valuable for these tasks.

SDSL EPR is predominantly performed by introducing one or more cysteines via site-directed mutagenesis and attaching a thiol-reactive spin label reagent such as S-(1-oxyl-2,2,5,5-tetramethyl-2,5-dihydro-1H-pyrrol-3-yl) methyl methanesulfonothioate (MTSL), yielding the spin-labeled side chain R1 [5]. While R1 is the most popular spin label, several alternatives exist that offer different reaction chemistries, linker lengths, chemical stability, and other properties that may be desirable depending on the experiment [32,33]. All data gathered from SDSL EPR experiments are necessarily co-determined by the spin label structure, dynamics, and environment in addition to the structure and dynamics of the protein they are attached to. Therefore, to obtain quantitative information about protein structure and dynamics from SDSL EPR data, it is crucial to accurately model the local structure and dynamics of the spin label.

To date, several spin label and protein modeling applications have been developed to aid in experimental design, interpretation, and protein modeling with SDSL EPR data [34–49]. While these methods generally perform well, it is currently difficult to use them with novel protein modeling protocols, integrate them with existing protein modeling software, or utilize them with novel spin labels. Several of these applications only allow for prediction of distance distributions and cannot be used to predict other types of experimental results such as membrane depth or solvent accessibility [37,40,43,50]. Of the available software that predict distance distributions, only a small number provide predefined docking or conformational change algorithms [34,45–48] that can only be minimally altered by the user. Integration with third-party modeling applications is often severely limited due to a lack of a scriptable interface. Additionally, most of these packages only implement one or a few spin labels and do not offer the ability to add new spin labels easily. Recently, significant efforts have been made to make spin label modeling more integrable and scriptable [49]. A spin label modeling package that integrates well with other protein modeling packages and allows users to easily add their own spin labels would aid investigators in the development of novel modeling protocols and utilization of cutting-edge protein modeling methods with SDSL restraints. These advancements would aid development, validation, and refinement of protein models, as well as the ability to expand these models to include alternate conformational states.

Here we introduce chiLife, a scriptable SDSL modeling package designed as a tool to develop novel SDSL EPR modeling and analysis pipelines. chiLife models spin labels on proteins, providing direct access to all the methods and properties of the spin labels, as well as allowing users to easily implement custom spin labels. chiLife is written in Python and thus can be integrated with the wide variety of Python-based protein modeling and analysis applications, such as MDAnalysis [51], PyRosetta [52], and Xplor-NIH [53,54]. In addition to EPR applications, chiLife can be used for other experiments such as paramagnetic relaxation enhancement (PRE) [55,56] or electron–nuclear double resonance (ENDOR) [57]. Below we provide an overview of the core functionality of chiLife and demonstrate several possible use cases through examples.

## 2. Design and implementation

**Fig 1:**
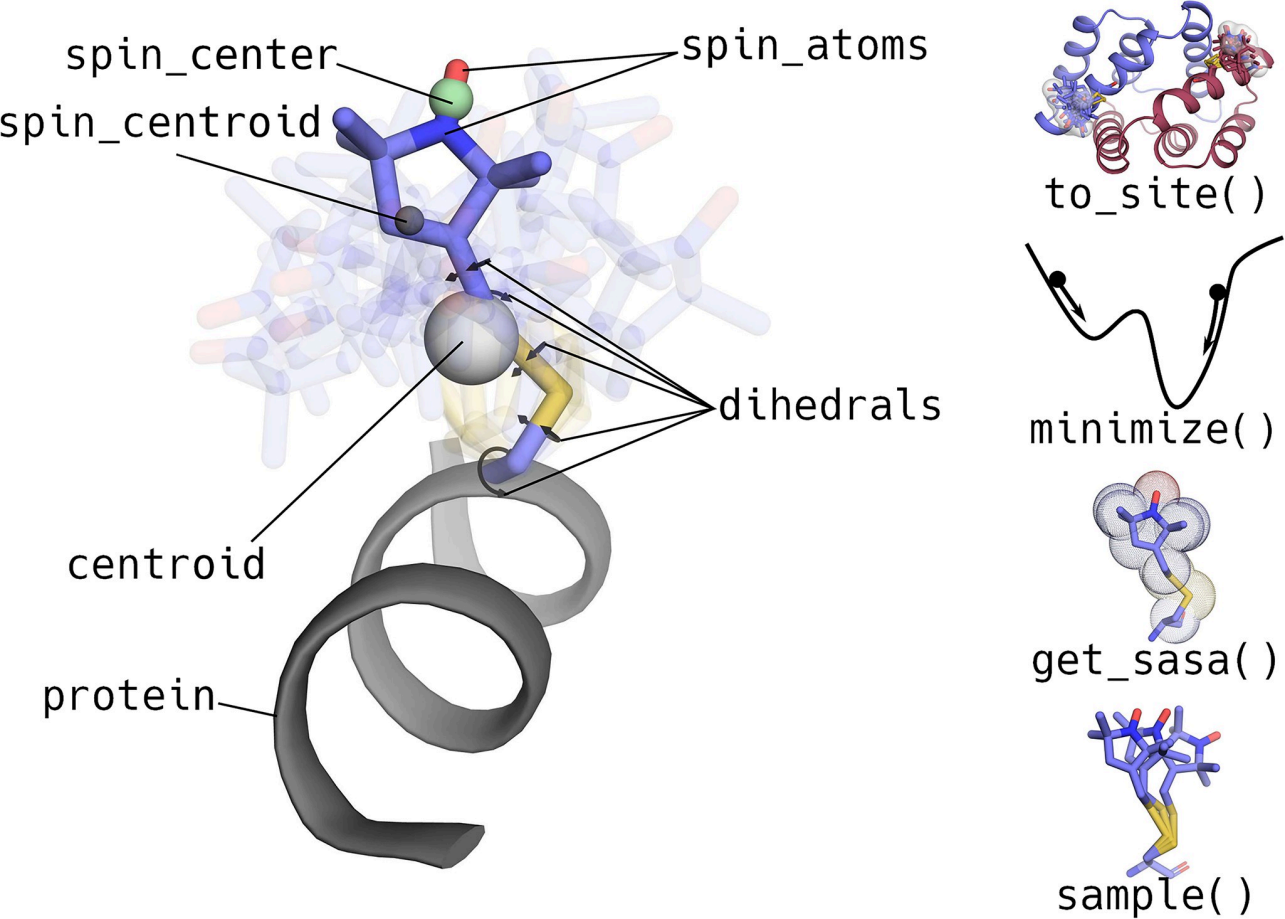
Illustration of chiLife’s SpinLabel object. On the left, some of its user-accessible properties are shown. On the right, some useful methods are illustrated that allow users to modify or calculate new structures from the SpinLabel object.

The central entities in chiLife are SpinLabel objects (Fig 1). A SpinLabel is derived from the parent RotamerEnsemble object, which represents a weighted ensemble of side chain rotamers aligned to a backbone site. Rotamers are represented by sets of values of their mobile dihedral angles (dihedrals), associated with weights (weights). Each dihedral angle can optionally be normally distributed, with separate standard deviations (dihedral_sigmas). Bond lengths and bond angles are fixed. The SpinLabel object extends the RotamerEnsemble object by adding the unpaired electron spin density, which is represented as a weighted distribution over one or more spin-bearing atoms (spin_atoms, spin_weights, spin_coords). This allows chiLife to represent labels with fully localized spin density (such as trityls, Cu^2+^, Gd^3+^) as well as with small delocalization (nitroxides) and extensive delocalization (triplet states of porphyrins [58]). The centers of the delocalized spin density of all rotamers are obtainable by the spin_centers property. These properties, along with many others, can be used to simulate experimental observables, validate and restrain computational models, and aid in the development of efficient and accurate experimental designs.

A SpinLabel object is usually created in the context of a protein by loading a rotamer library and attaching it to a protein structure. To interact with protein models, chiLife uses the widely used MDAnalysis library [51]. This allows users to make use of the rich features and atom selection language offered by MDAnalysis. A SpinLabel is attached to a target protein site by aligning the rotamers from the rotamer library and target site by a method called bisect. This method translates the rotamers to superimpose their CA atoms with the CA of the protein and rotates the rotamers such that the N–CA–C planes and the vectors bisecting the N–CA–C angles are aligned. This alignment method assures that the side chain atoms are not biased to one side of the residue in the case that the rotamer library and the protein target site have different N–CA–C angles. Other alignment methods are implemented and selectable during SpinLabel construction. If the sample = n argument is given, where n is a user-defined number of samples, a set of off-rotamer samples [59] is generated and attached instead of the rotamer library. The degree of off-rotamer sampling is controlled by the rotamer library being used but can be overridden by the dihedral_sigmas keyword argument. If dihedral_sigmas is set to numpy.inf then the sampling method effectively samples the entire volume accessible to the spin label side chain [36,45,46]. Next, clashes between the sampled rotamers and surrounding side chains are evaluated using a flat-top repulsive Lennard–Jones potential energy function, and rotamers with high predicted total energy and consequently low population are trimmed from the ensemble. A forgive factor for the Lennard–Jones potential as well as a maximum distance for clash evaluations can be provided. The energy function used can be modified by providing an alternate built-in or a user-defined energy function via the energy_func keyword argument. Clash evaluations can be turned off via the eval_clash keyword argument.

Once attached, a spin label can be further manipulated or used for analysis as illustrated in Fig 1. Summary quantities such as centroid, the centroid of all the heavy atoms of the attached ensemble, and spin_centroid, the centroid of the spin_centers of all rotamers in the ensemble, can be used when modeling systems with aggregate measurements like membrane depth. The SpinLabel object gives users control over how rotamer ensembles are constructed, how energies are evaluated, and how the ensembles are manipulated, while offering practical defaults and access to attributes that can be used to predict experimental observables.

## 3. Results

### 3.1. Basic spin label modeling

**Fig 2:**
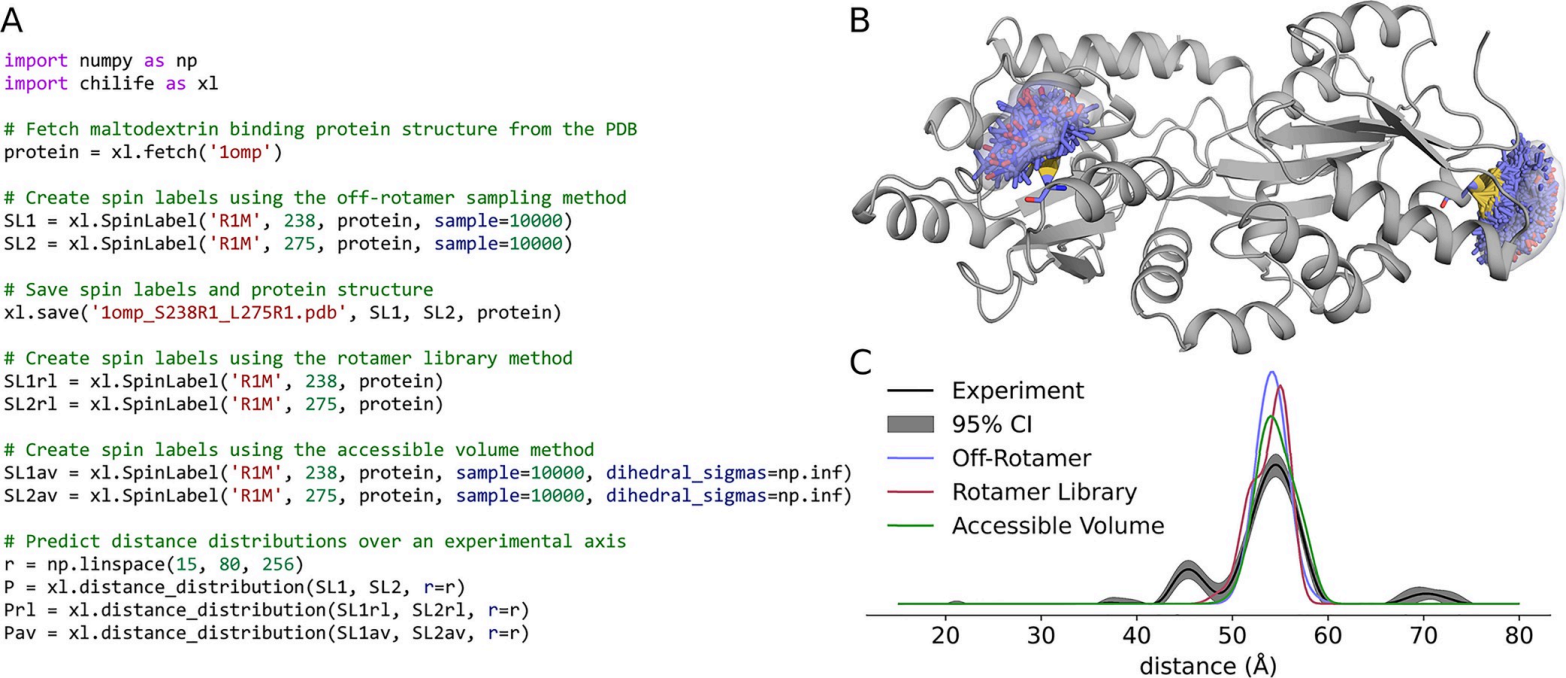
Spin labeling proteins and predicting spin label distance distributions. A) chiLife script. B) Cartoon model of maltose binding protein (PDBID 1OMP) labeled with R1 at sites 238 and 275, showing the spin label ensembles (sticks) and weighted kernel density estimates of the spin centers (semitransparent surfaces). C) Comparison of the predicted distance distributions with the experimental distance distribution.

chiLife has a range of built-in methods for modeling spin labels on proteins. The first example illustrates three of them: the rotamer library (RL) method [34,41], used if the sample keyword argument is not set, the accessible-volume (AV) method [36,45], accessed by setting sample to an integer and dihedral_sigmas to numpy.inf, and an off-rotamer sampling method [59] that is controlled by sample and dihedral_sigmas arguments. These methods are illustrated in the script shown in Fig 2A. In this example, a protein object is created by fetching a structure of maltodextrin/maltose binding protein (MBP) from the PDB [60]. The fetch function returns an MDAnaysis Universe object which contains all chains, states, and ligands of the fetched PDB. Users can utilize the MDAnalysis select_atoms function to create an AtomGroup containing only the chains, ligands, or atoms of interest [51]. When provided with an AtomGroup, chiLife will only consider these atoms when evaluating external interactions, except for water molecules which are implicitly excluded. The exclusion of any other atoms must be managed by the user. Next, SpinLabel objects are constructed for two sites using the three different methods described above. The first two spin label ensembles and the protein structure are saved to a new PDB file for visualization. Finally, distance distributions are generated from each pair of SpinLabel objects using the distance_distribution function. This function accepts an arbitrary number of spin labels and returns the cumulative distribution from all spin label pairs. The RL and AV methods were initially implemented in the applications MMM [34,41] and MTSSLWizard [36,45] respectively, and while chiLife supports them, most default parameters are not the same. Thus, to be able to accurately replicate results from these packages, chiLife offers the from_mmm and from_wizard class methods.

Fig 2B plots the spin label objects as stored in the PDB file. This file contains an unaltered copy of the protein (if provided), all the spin labels as separate multistate models with their relative populations stored as occupancy factors, and a set of pseudo-atom coordinates representing the spin centers of the spin label rotamers. These pseudo-atoms are shown as a surface in Fig 2B with the relative populations mapped to the color of the surface. Fig 2C shows the distance distributions predicted by the three labeling methods and compares them to the experimentally determined distribution [59]. All three methods provide consistent prediction of a predominant distance at about 55 Å. The smaller peak at about 45 Å likely is due to a subpopulation of MBP in the maltose-bound conformation that is known to be sampled in the absence of maltose [56].

### 3.2. SDSL screen of solvent-accessible surface residues

One important application of *in silico* spin label modeling is the use of site pair scans to predict distance distributions for designing experiments that test protein models. With chiLife, a script can be used for screening solvent-exposed site pairs of proteins in order to find the optimal spin labeling sites to investigate conformational changes or to obtain experimental evidence that best discerns between competing protein models. Listing S1 in S1 Text performs a screen of all solvent-accessible surface residues (>50 Å^2^ solvent accessible surface) of MBP in the apo and holo state for maximal distance contrast. This is done by modeling spin labels at all sites and predicting pairwise distance distributions between all labels for both states. Then the predicted distributions are screened for site pairs that distinguish the bound (PDBID: 1ANF) and unbound (PDBID: 1OMP) states of MBP, in this case by maximizing the earth-mover’s distance between the two distributions.

**Fig 3:**
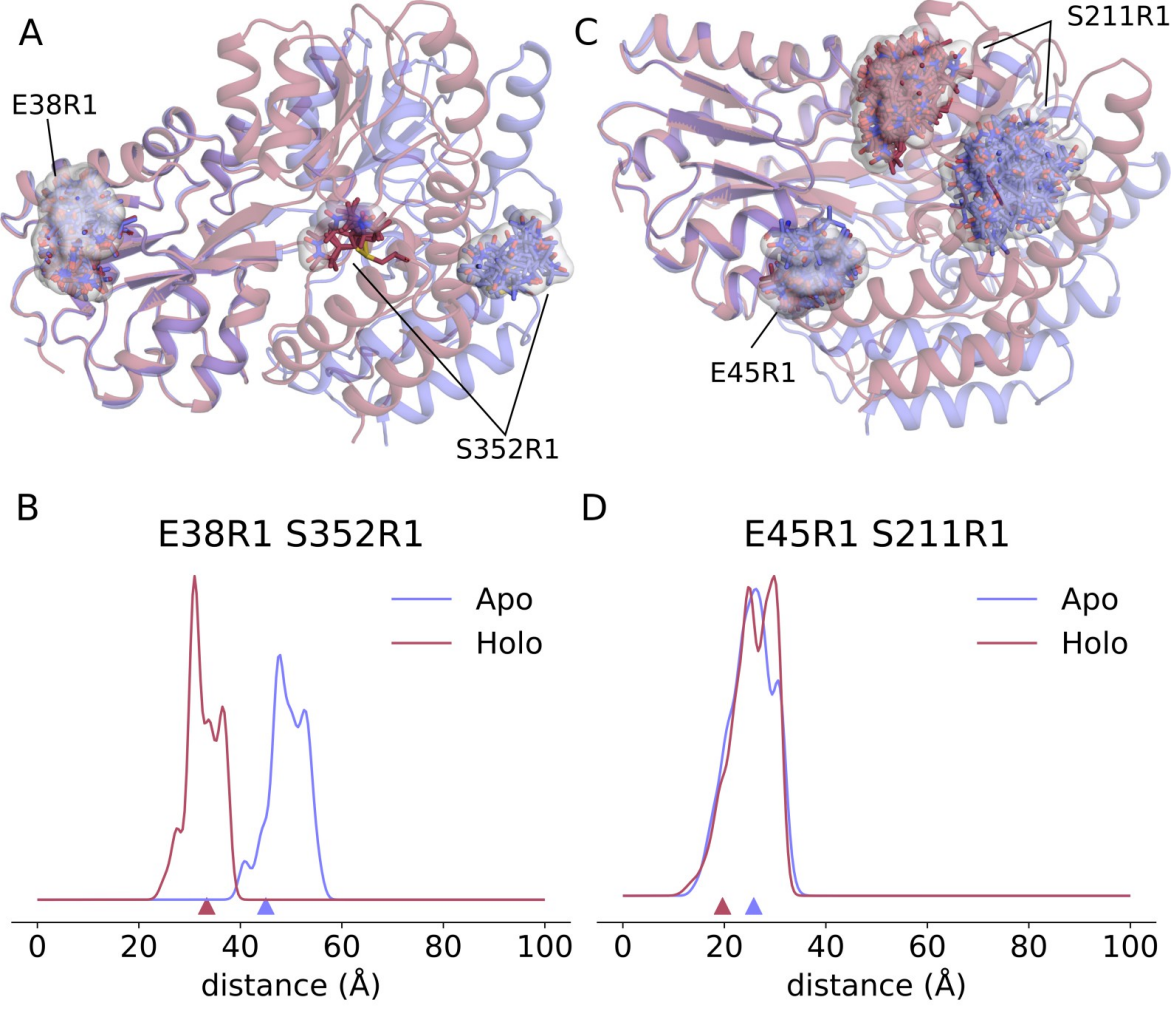
Illustration of how changes in backbone do not necessarily cause changes in distance distributions. Top: Comparison of apo (blue, PDBID 1OMP) and holo (red, PDBID 1ANF) MBP structures and the locations of the model R1 spin labels (sticks) for the site pairs E38R1 S352R1 (A) and E45R1 S211R1 (B). Bottom: Comparison of apo and holo distance distributions for the two site pairs that both show significant changes in Cβ–Cβ backbone distance, indicated by small triangles at the base of the plots. The E38R1 S352R1 site pair on the left (C) shows a clear difference in the predicted distributions while the E45R1 S211R1 site pair on the right (D) shows very little change.

Fig 3A shows the spin labels of the best site pair (residues 38 and 352) attached to the two conformations of MBP and Fig 3B shows the two predicted distance distributions. This type of analysis facilitates designing the optimal SDSL site pairs when investigating protein conformational change. In some cases, this may be superior to screening for a change in protein backbone distance alone since it considers the relative orientation and rotamer distribution of the spin labels. To illustrate this, Fig 3C and 3D show a site pair (residues 45 and 211) with a significant change in the backbone Cβ–Cβ distance, but little change in the spin label distance distribution.

### 3.3. Adding custom spin labels

**Fig 4:**
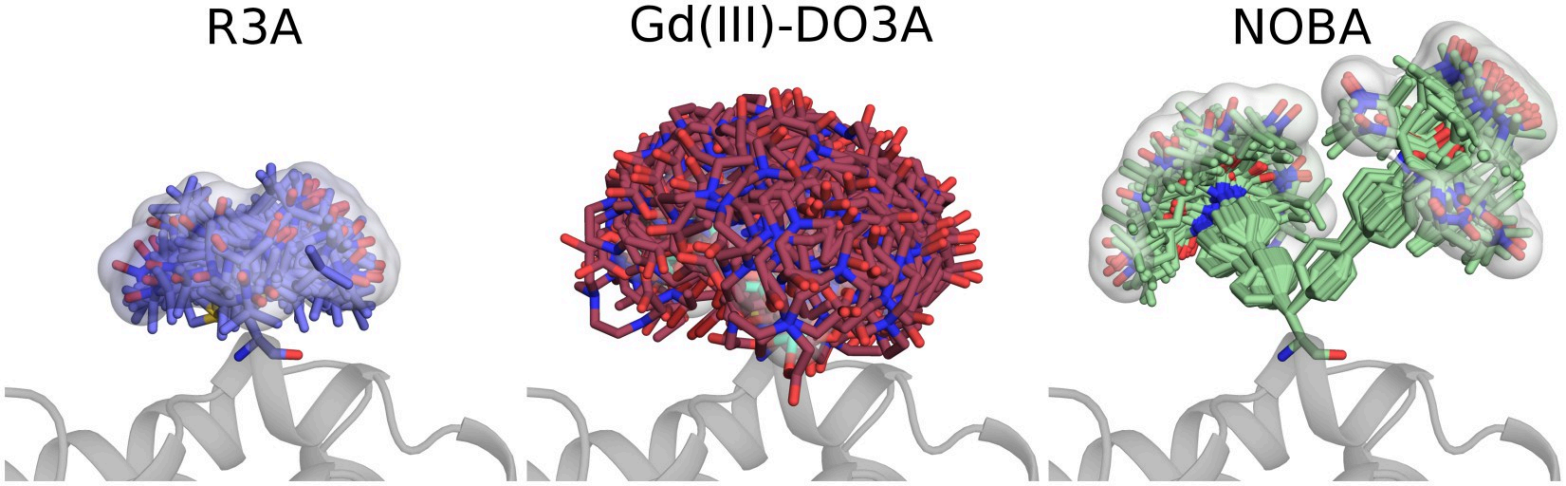
Three spin labels added to chiLife and attached to T4 lysozyme (PDBID: 2LZM) at site 109. R3 (left, sticks with blue carbons) is a small highly mobile nitroxide label. Gd(III)-DO3A (center, sticks with dark red carbons) is a gadolinium-based spin label resistant to reduction. NOBA (right, sticks with green carbons) is a biorthogonal nitroxide. The surface is made by pseudo-atoms at the rotamer spin centers.

In addition to chiLife’s built-in rotamer libraries for common spin labels, users can add rotamer libraries for new spin labels. This is important as new spin labels are continuously being developed for various applications featuring bio-orthogonal coupling chemistry [61–63], shorter linker lengths [64,65], enhanced phase memory times [66], and resistance to chemical reduction [67]. Listing S2 in S1 Text creates new chiLife rotamer libraries for the three spin labels R3A [68], Gd(III)-DO3A [69] and NOBA [70] using multistate PDB files. Fig 4 illustrates the use of the new rotamer libraries on T4 lysozyme.

The rotamer libraries consist of a set of low-energy conformers and their associated internal energies generated using CREST [71]. Each new rotamer library is created using the create_library function, which allows the user to specify the mobile dihedral angles as a list of quadruplets, and the atoms and fractional populations for the unpaired electron spin density. The rotamers of the library can also be weighted by providing an array or list of weights via the weights argument.

The create_library function is very versatile and can create libraries from a single-state or multi-state PDB file. It can create libraries that contain only one structure that can randomly sample new dihedral angles, and libraries that have multiple rotameric states but no independent rotatable side chain dihedral angles such as 2,2,6,6-tetramethyl-N-oxyl-4-amino-4-carboxylic acid (TOAC). Additionally, when using a multi-state PDB file, chiLife will retain any stereoisomeric heterogeneity present among the states in the file. This feature is particularly useful for labels with reaction chemistries which create diastereomers when reacting with thiols, such as maleimides.

The output of the create_library function is a file in NPZ format which can be used by specifying the rotlib keyword argument when constructing a SpinLabel. These rotamer library files can be shared with coworkers and collaborators. The rotamer library name is distinct from the residue name, allowing the use of multiple rotamer libraries for the same residue type, which can aid in rotamer library development and benchmarking.

### 3.4. Local side chain repacking

**Fig 5:**
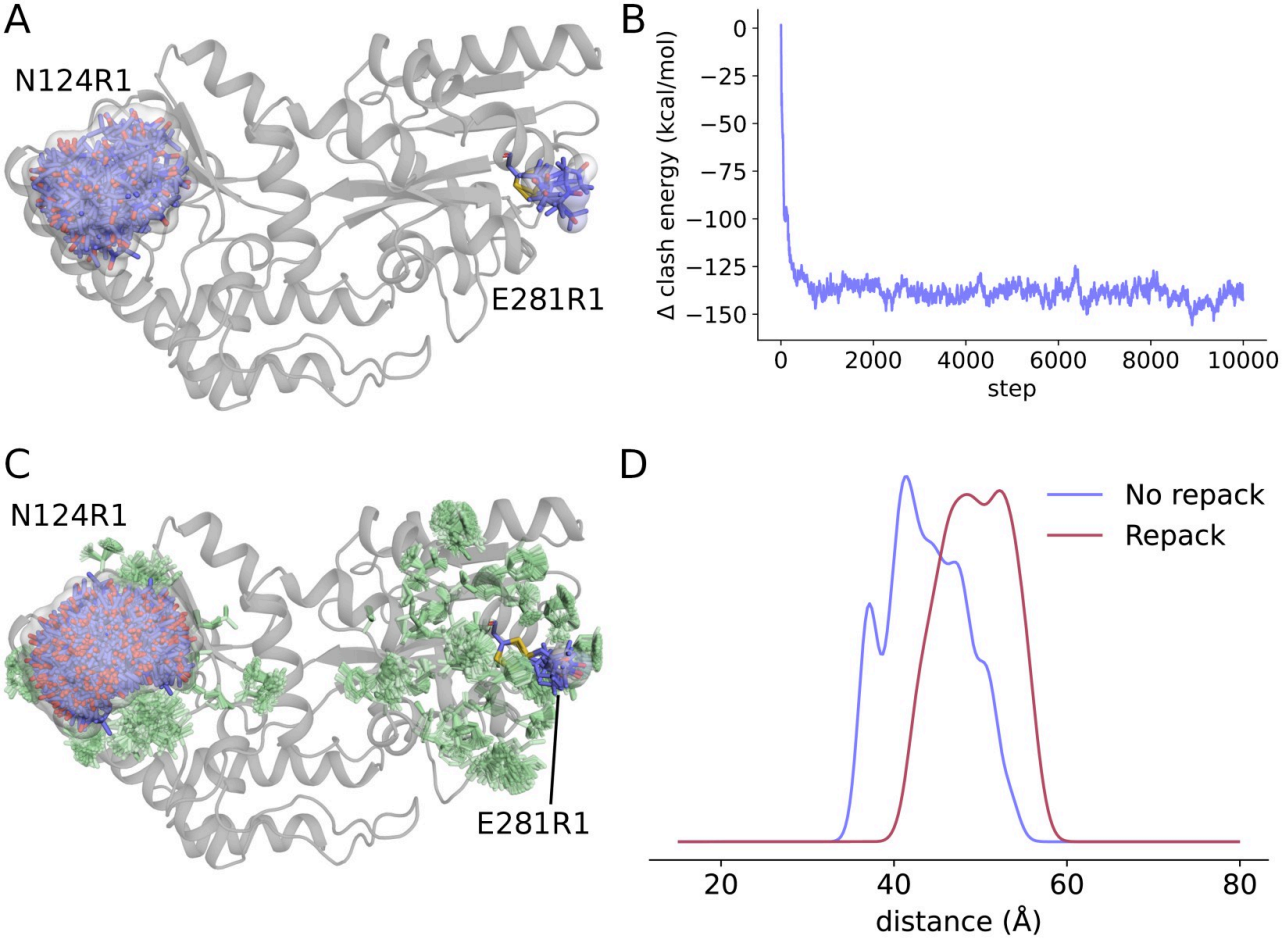
Local side chain repacking for R1 spin label ensembles at sites N124R1 and E281R1 on MBP (PDBID 1OMP). A) Prediction of spin label ensembles without repacking. The protein structure is shown as a gray cartoon. Spin labels are shown as blue sticks. B) Energy trajectory of MCMC repacking, relative to the energy of the starting structure. C) Spin label (blue sticks) and neighboring side chain (green sticks) ensembles obtained from the repacking trajectory. D) Comparison of predicted distance distributions derived from the ensembles of the repacked and the original structures.

When modeling spin labels very close together or in crowded environments, it might be important to model changes in the conformations of neighboring side chains as well by performing local side chain repacking. This can be accomplished using the chiLife repack function, as illustrated in Listing S3 in S1 Text and Fig 5. The repack function uses Markov chain Monte Carlo (MCMC) sampling to repack [72] all residues within a user-defined radius of a set of SpinLabel objects. To do this, chiLife relies on the widely used Dunbrack rotamer libraries [73] for canonical amino acid side chains. In each MCMC sampling step, a spin label or neighboring site is chosen at random and a new rotamer is sampled from the rotamer library associated with that site. If the off_rotamer option is set to True, new off-rotamer dihedrals are sampled for that rotamer. The step is accepted or rejected using the Metropolis–Hastings criterion based on energy. The outputs of the repack function are an MDAnalysis Universe object of the protein structures for all the accepted steps and a list of the relative energies for all steps. From this trajectory, the from_trajectory method builds a new SpinLabel object (neglecting a user-adjustable number of initial burn-in steps), which can then be used in the same way as any other SpinLabel object in chiLife.

Fig 5 illustrates the effect, and potential benefit, of repacking. Fig 5A shows MBP labeled at two sites using the ORS method. Despite ORS, the rotamer ensembles are still confined by the rigid neighboring residues. Fig 5B shows the trajectory of the energy score function during MCMC repacking. It illustrates a rapid improvement in energy during the first 1000 steps, followed by a long steady-state sampling of spin label and neighboring amino acid side chain conformations. Fig 5C shows the results of this repacking, and Fig 5D plots the predicted distance distributions derived from the original and repacked structures. The repacked structure shows a distance distribution that is significantly different from the one made without repacking.

### 3.5. Membrane docking

**Fig 6:**
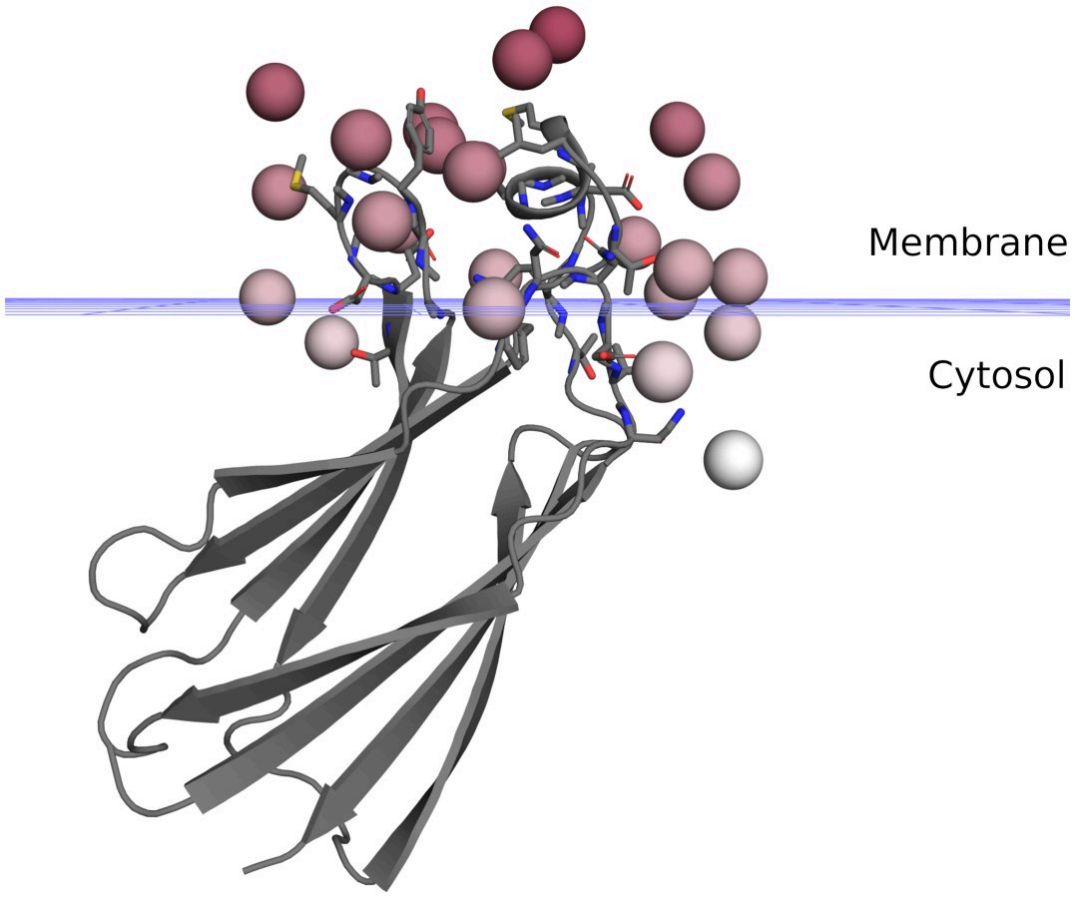
Membrane docking of the cPLA2 C2 domain (PDBID 1BCI). The cPLA2 C2 domain is shown as a gray cartoon. Spin centroids are shown as red spheres with their color saturation mapped to the experimental depth. Native side chains of spin labeled sites are shown as sticks. The blue grid indicates the phosphate plane of the model.

While EPR excels at measuring ensemble distance distributions between spin labels, it is also useful for providing data to answer other questions such as how and where proteins interact with membranes [6]. With chiLife, users can accomplish modeling tasks driven by many types of SDSL EPR data. As an example, Listing S4 in S1 Text utilizes spin label membrane depth data from Malmberg et al. [8] to determine the position and orientation of the C2 domain of cytosolic phospholipase A2 (cPLA2) in the membrane. These data are calculated from power saturation EPR measurements of spin labels on several sites in the presence of relaxation agents with different membrane permeability. For each site, a SpinLabel object is created, and its spin_centroid, the weighted average position of the spin_atoms over the whole rotamer ensemble, is determined. Then, a least-squares fit is performed to determine the Z-position of the protein and the three Euler angles describing its orientation by minimizing the difference between the Z-coordinates of the centroid and the experimentally determined depth. The resulting model, shown in Fig 6, is in good agreement with previously reported models [8,74]. This approach takes full advantage of the spin label modeling methods available in chiLife and does not require manual modeling of rigid rotamers as performed previously [8,74], nor does it require mutating the original amino acids. Similar protocols can be developed to dock or orient other membrane-associated proteins in a lipid bilayer. Additionally, modelling protocols for other SDSL EPR experiments can be developed making use of solvent accessibility and spin label mobility data [75,76].

### 3.6. Custom scoring functions

**Fig 7:**
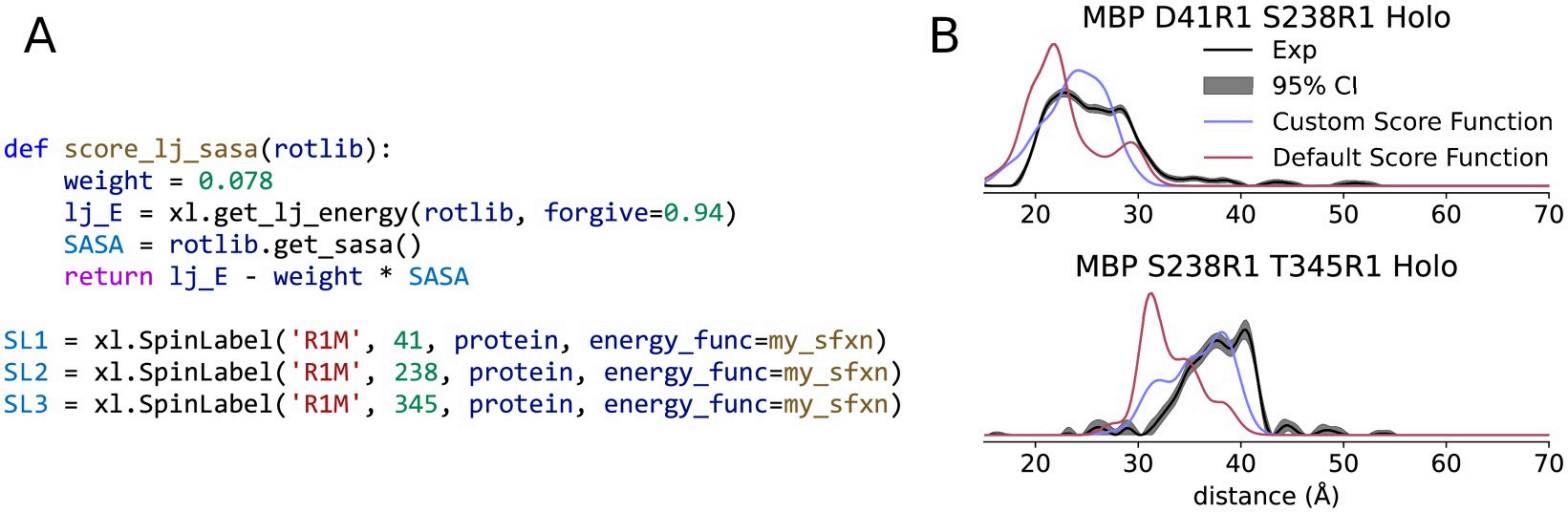
Comparison of distance distribution predictions using different scoring functions. A) Definition and application of custom energy function using chiLife. B) Experimental distance distributions for two site pairs of MPB, taken from [59], are compared to the predicted distance distributions from spin labels modeled using a modified Lennard–Jones potential and a custom function that augments the same potential with an additional term to account for compensatory attractive forces with the solvent.

In addition to custom modeling protocols, chiLife allows users to define and utilize their own energy functions, either independently developed or interfaced from other molecular modeling methods. The only requirement for an energy function is that it accepts a RotamerEnsemble or SpinLabel object and outputs an energy for each rotamer in the ensemble in kcal/mol. Fig 7 provides an example where the scoring function, consisting of a modified Lennard–Jones potential with a forgive factor and a maximum energy cap, is augmented with an additional attractive term proportional to the solvent accessible surface area of the rotamer. This term is meant to capture the van der Waals forces between the solvent and solvent-accessible surface atoms of the rotamers. The motivation for this is that the attractive force contributions of the Lennard–Jones potential can bias the rotamers to form intramolecular interactions with the surface of the protein if the compensatory van der Waals interactions with solvent molecules are neglected [77]. The forgive factor and the weight of the SASA term were fitted to produce the best agreement with a recently published DEER data set for MBP [59]. Fig 7 shows how this custom scoring function performs on two previously published constructs of MBP [59] which use highly solvent-accessible sites. For these data, the SASA-augmented score function results in significant improvements in the distance distribution prediction accuracy compared to the modified Lennard–Jones potential alone.

While this example illustrates the customizability of chiLife, it also illustrates how chiLife can be used in an exploratory fashion to further develop spin label modeling methods. Replacing the custom score function with methods from third-party software allows to integrate novel developments in other fields into spin label modeling. For example, use of the general forcefield offered by xTB has recently shown promise as a powerful tool for modeling spin labels, because of its high-resolution score function and its rapid and accurate parametrization of spin labels, which are often difficult to parametrize for traditional force fields [78,79]. Similarly, deep-learning potentials [80,81] show promise as high-resolution score functions for spin label modeling.

### 3.7. Interaction with third-party software: Rosetta

**Fig 8:**
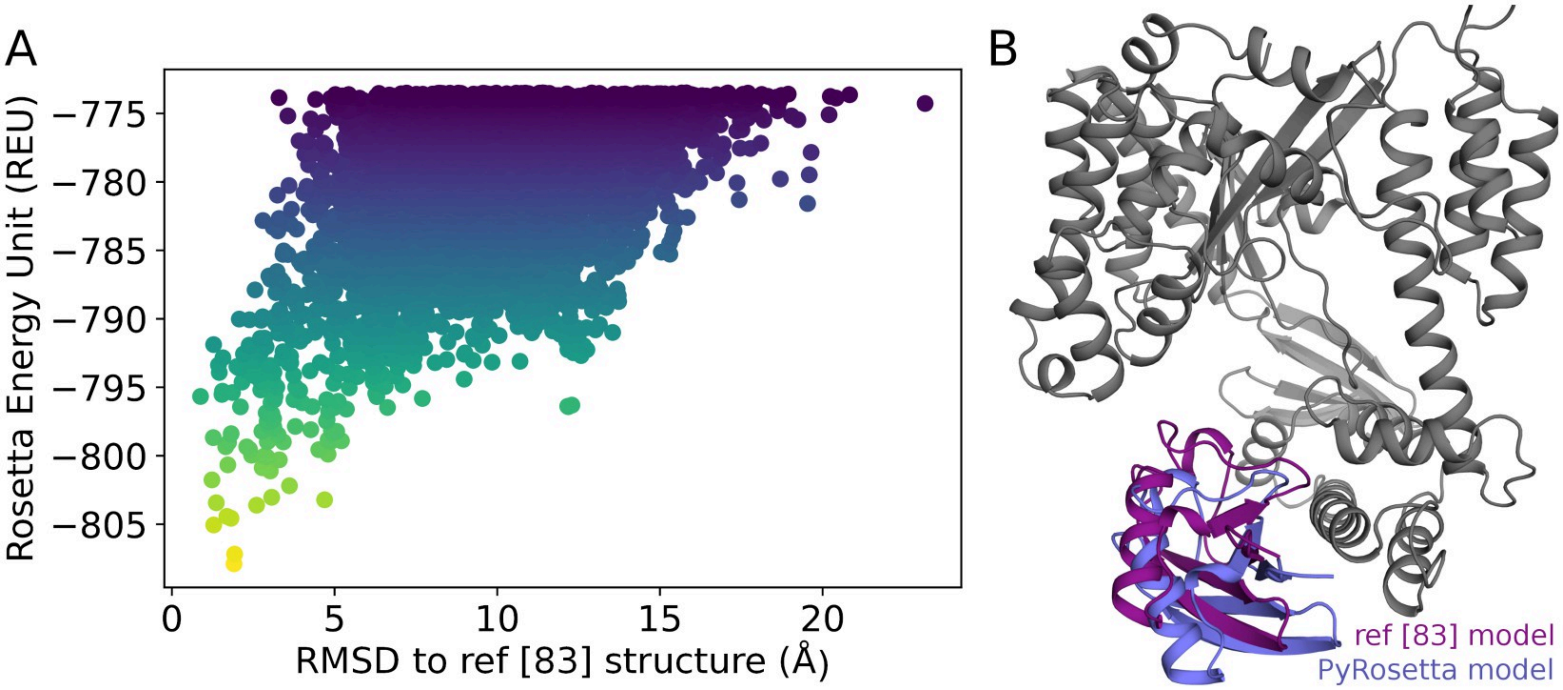
Comparison of a previously published ExoU–ubiquitin complex model and the best scoring model obtained by integrating chiLife and PyRosetta. A) Docking funnel showing convergence towards the previously published complex using chiLife restraints. B) Cartoon structures of ExoU (gray) showing predicted locations of ubiquitin in the previously published model (purple) and in the PyRosetta model produced here (blue).

Because chiLife is written in Python and designed for modular modeling pipelines, it can be integrated with other Python-based molecular modeling packages. This integration can be used to aid chiLife in modeling spin labels as discussed above, or to use chiLife to aid protein modeling. Listing S5 in S1 Text is a PyRosetta [52] script illustrating how chiLife can be integrated with PyRosetta. Using previously published DEER data obtained for nine site pairs [82,83], it models the interaction between the bacterial protein toxin ExoU and its cofactor ubiquitin [82–84]. Fig 8 compares the model obtained using PyRosetta and chiLife with the previously published and experimentally validated model [83]. Fig 8A shows docking funnels that confirm convergence of the algorithm towards the previously published structure. Fig 8B compares the two models [83]. Both models have a hydrophobic patch of ubiquitin interfacing with the same hydrophobic residues of ExoU α-helix 18, with a Cα root mean square deviation (RMSD) of less than 2 Å.

This example illustrates how chiLife can be integrated with other protein modeling Python packages. Notably, Rosetta has some built-in utilities for modeling proteins with the R1 spin label [38,39]; however, the data used here include some DEER experiments conducted with the brominated spin label R7, which produces different distance distributions than R1 [85]. While R1 only differs from R7 by one atom (H vs. Br), this approach is readily applicable to all spin labels supported by chiLife as well as custom spin labels. Additionally, any of the previously published spin label modeling methods can be used, including the rotamer library approach [41], the accessible volume approach [36] and the off-rotamer sampling approach [59]. Because of the diversity and modularity of PyRosetta, the scoring term used in this example can also be used to perform other modeling tasks such as structural refinement, *ab initio* folding, or conformational change modeling.

## 4. Availability and future directions

SDSL EPR is a powerful integrative method for probing protein structure and dynamics. To aid these investigations, we have developed chiLife, a modular, scriptable spin label modeling package that facilitates the rapid development of application-specific protein modeling pipelines using SDSL EPR data. We described several examples that illustrate how chiLife can be used for experimental design, analysis, and protein modeling. As protein modeling methods are outpacing experimental structure determinations, the utility of integrative methods like SDSL EPR will become invaluable for model validation, refinement, and hypothesis development. chiLife will support these endeavors by offering flexible spin label modeling methods that can be integrated into custom modeling or analysis workflows.

chiLife is a free and open-source Python API and is available from https://github.com/StollLab/chiLife. A stable version of chiLife can be installed using the pip package manager by running pip install chiLife. A development version can be installed from GitHub using the instructions available on the GitHub page.

## Supporting information

S1 Text Supplementary Text. (PDF)

## References

[1] Jeschke G. The contribution of modern EPR to structural biology. Emerg Top Life Sci. 2018;2: 9–18. doi: 10.1042/ETLS20170143 33525779 PMC7288997

[2] Sahu ID, Lorigan GA. Biophysical EPR studies applied to membrane proteins. J Phys Chem Biophys. 2015;6: 188. doi: 10.4172/2161-0398.1000188 26855825 PMC4742357

[3] Hubbell WL, Cafiso DS, Altenbach C. Identifying conformational changes with site-directed spin labeling. Nat Struct Mol Biol. 2000;7: 735. doi: 10.1038/78956 10966640

[4] Klug CS, Feix JB. Methods and applications of site-directed spin labeling EPR spectroscopy. Methods Cell Biol. 2008;84: 617–658. doi: 10.1016/S0091-679X(07)84020-9 17964945

[5] Altenbach C, Flitsch SL, Khorana HG, Hubbell WL. Structural studies on transmembrane proteins. 2. Spin labeling of bacteriorhodopsin mutants at unique cysteines. Biochemistry. 1989;28: 7806–12. doi: 10.1021/bi00445a042 2558712

[6] Altenbach C, Greenhalgh D, Khorana H, Hubbell W. A collision gradient method to determine the immersion depth of nitroxides in lipid bilayers: application to spin-labeled mutants of bacteriorhodopsin. Proc Natl Acad Sci USA. 1994;91: 1667–71. doi: 10.1073/pnas.91.5.1667 8127863 PMC43224

[7] Altenbach C, Froncisz W, Hemker R, Mchaourab H, Hubbell WL. Accessibility of nitroxide side chains: absolute Heisenberg exchange rates from power saturation EPR. Biophys J. 2005;89: 2103–12. doi: 10.1529/biophysj.105.059063 15994891 PMC1366712

[8] Malmberg NJ, Buskirk DR, Falke JJ. Membrane-docking loops of the cPLA2 C2 domain: Detailed structural analysis of the protein−membrane interface via site-directed spin-labeling. Biochemistry. 2003;42: 13227–13240. doi: 10.1021/bi035119+ 14609334 PMC3637888

[9] Tessmer MH, Anderson DM, Buchaklian A, Frank DW, Feix JB. Cooperative substrate-cofactor interactions and membrane localization of the bacterial phospholipase A2 (PLA2) enzyme, ExoU. J Biol Chem. 2017;292: 3411–3419. doi: 10.1074/jbc.M116.760074 28069812 PMC5336173

[10] Sato H, Feix JB. Peptide–membrane interactions and mechanisms of membrane destruction by amphipathic α-helical antimicrobial peptides. Biochim Biophys Acta, Biomembranes. 2006;1758: 1245–1256. doi: 10.1016/j.bbamem.2006.02.021 16697975

[11] Jeschke G. DEER distance measurements on proteins. Annual review of physical chemistry. 2012;63: 419–46. doi: 10.1146/annurev-physchem-032511-143716 22404592

[12] Torricella F, Pierro A, Mileo E, Belle V, Bonucci A. Nitroxide spin labels and EPR spectroscopy: A powerful association for protein dynamics studies. Biochimica et Biophysica Acta (BBA)—Proteins and Proteomics. 2021; 140653. doi: 10.1016/j.bbapap.2021.140653 33757896

[13] Glaenzer J, Peter MF, Hagelueken G. Studying structure and function of membrane proteins with PELDOR/DEER spectroscopy–The crystallographers’ perspective. Methods. 2018;147: 163–175. doi: 10.1016/j.ymeth.2018.03.002 29510248

[14] Pannier M, Veit S, Godt A, Jeschke G, Spiess HW. Dead-time free measurement of dipole-dipole interactions between electron spins. J Magn Reson. 2000;142: 331–40. doi: 10.1006/jmre.1999.1944 10648151

[15] Martin RE, Pannier M, Diederich F, Gramlich V, Hubrich M, Spiess HW. Determination of end to end distances in a series of TEMPO diradicals of up to 2.8 nm length with a new four pulse double electron electron resonance experiment. Angew Chem Int Ed Engl. 1998;37: 2833–2837. 29711097 10.1002/(SICI)1521-3773(19981102)37:20<2833::AID-ANIE2833>3.0.CO;2-7

[16] Puljung MC, DeBerg HA, Zagotta WN, Stoll S. Double electron-electron resonance reveals cAMP-induced conformational change in HCN channels. Proc Natl Acad Sci USA. 2014;111: 9816–21. doi: 10.1073/pnas.1405371111 24958877 PMC4103371

[17] Evans EGB, Morgan JLW, DiMaio F, Zagotta WN, Stoll S. Allosteric conformational change of a cyclic nucleotide-gated ion channel revealed by DEER spectroscopy. Proc Natl Acad Sci USA. 2020;20: 10839–10847. doi: 10.1073/pnas.1916375117 32358188 PMC7245078

[18] Tessmer MH, DeCero SA, del Alamo D, Riegert MO, Meiler J, Frank DW, et al. Characterization of the ExoU activation mechanism using EPR and integrative modeling. Sci Rep. 2020;10: 19700. doi: 10.1038/s41598-020-76023-3 33184362 PMC7665212

[19] del Alamo D, Jagessar KL, Meiler J, Mchaourab HS. Methodology for rigorous modeling of protein conformational changes by Rosetta using DEER Distance Restraints. PLoS Comput Biol. 2021;17: e1009107. doi: 10.1371/journal.pcbi.1009107 34133419 PMC8238229

[20] Sala D, del Alamo D, Mchaourab HS, Meiler J. Modeling of protein conformational changes with Rosetta guided by limited experimental data. Structure. 2022;30: 1157–1168.e3. doi: 10.1016/j.str.2022.04.013 35597243 PMC9357069

[21] Dastvan R, Fischer AW, Mishra S, Meiler J, Mchaourab HS. Protonation-dependent conformational dynamics of the multidrug transporter EmrE. Proc Natl Acad Sci USA. 2016;113: 1220–5. doi: 10.1073/pnas.1520431113 26787875 PMC4747756

[22] Elgeti M, Hubbell WL. DEER analysis of GPCR conformational heterogeneity. Biomolecules. 2021;11: 778. doi: 10.3390/biom11060778 34067265 PMC8224605

[23] Lerch MT, Matt RA, Masureel M, Elgeti M, Kumar KK, Hilger D, et al. Viewing rare conformations of the β2 adrenergic receptor with pressure-resolved DEER spectroscopy. Proc Natl Acad Sci USA. 2020; 202013904. doi: 10.1073/pnas.2013904117 33257561 PMC7749303

[24] Timachi MH, Hutter CA, Hohl M, Assafa T, Böhm S, Mittal A, et al. Exploring conformational equilibria of a heterodimeric ABC transporter. Elife. 2017;6: e20236. doi: 10.7554/eLife.20236 28051765 PMC5216877

[25] Lerch MT, Yang Z, Brooks EK, Hubbell WL. Mapping protein conformational heterogeneity under pressure with site-directed spin labeling and double electron-electron resonance. Proc Natl Acad Sci USA. 2014;111: E1201–10. doi: 10.1073/pnas.1403179111 24707053 PMC3977274

[26] Jeschke G. Characterization of protein conformational changes with sparse spin-label distance constraints. J Chem Theory Comput. 2012;8: 3854–3863. doi: 10.1021/ct300113z 26593026

[27] Schmidt T, Wang D, Jeon J, Schwieters CD, Clore GM. Quantitative agreement between conformational substates of holo calcium-loaded calmodulin detected by double electron–electron resonance EPR and predicted by molecular dynamics simulations. J Am Chem Soc. 2022. doi: 10.1021/jacs.2c02201 35759799 PMC9359069

[28] del Alamo D, DeSousa L, Nair RM, Rahman S, Meiler J, Mchaourab HS. Integrated AlphaFold2 and DEER investigation of the conformational dynamics of a pH-dependent APC antiporter. Proc Natl Acad Sci USA. 2022;119: e2206129119. doi: 10.1073/pnas.2206129119 35969794 PMC9407458

[29] Baek M, DiMaio F, Anishchenko I, Dauparas J, Ovchinnikov S, Lee GR, et al. Accurate prediction of protein structures and interactions using a three-track neural network. Science. 2021;373: 871–876. doi: 10.1126/science.abj8754 34282049 PMC7612213

[30] Jumper J, Evans R, Pritzel A, Green T, Figurnov M, Ronneberger O, et al. Highly accurate protein structure prediction with AlphaFold. Nature. 2021;596: 583–589. doi: 10.1038/s41586-021-03819-2 34265844 PMC8371605

[31] Lin Z, Akin H, Rao R, Hie B, Zhu Z, Lu W, et al. Evolutionary-scale prediction of atomic level protein structure with a language model. bioRxiv. 2022; 2022.07.20.500902. doi: 10.1101/2022.07.20.500902 36927031

[32] Ackermann K, Chapman A, Bode BE. A comparison of cysteine-conjugated nitroxide spin labels for pulse dipolar EPR spectroscopy. Molecules. 2021;26: 7534. doi: 10.3390/molecules26247534 34946616 PMC8706713

[33] Braun TS, Widder P, Osswald U, Groß L, Williams L, Schmidt M, et al. Isoindoline-based nitroxides as bioresistant spin labels for protein labeling through cysteines and alkyne-bearing noncanonical amino acids. Chembiochem. 2020. doi: 10.1002/cbic.201900537 31657498 PMC7187341

[34] Jeschke G. MMM: A toolbox for integrative structure modeling. Protein Sci. 2018;27: 76–85. doi: 10.1002/pro.3269 28799219 PMC5734387

[35] Alexander NS, Stein RA, Koteiche HA, Kaufmann KW, Mchaourab HS, Meiler J. RosettaEPR: Rotamer library for spin label structure and dynamics. PLoS One. 2013;8: e72851. doi: 10.1371/journal.pone.0072851 24039810 PMC3764097

[36] Hagelueken G, Ward R, Naismith JH, Schiemann O. MtsslWizard: In silico spin-labeling and generation of distance distributions in PyMOL. Appl Magn Reson. 2012;42: 377–391. doi: 10.1007/s00723-012-0314-0 22448103 PMC3296949

[37] Beasley KN, Sutch BT, Hatmal MM, Langen R, Qin PZ, Haworth IS. Computer modeling of spin labels NASNOX, PRONOX, and ALLNOX. Methods Enzymol. 2015;563: 569–593. doi: 10.1016/bs.mie.2015.07.021 26478499

[38] Hirst SJ, Alexander N, Mchaourab HS, Meiler J. RosettaEPR: an integrated tool for protein structure determination from sparse EPR data. J Struct Biol. 2011;173: 506–14. doi: 10.1016/j.jsb.2010.10.013 21029778 PMC3040274

[39] Alamo DD, Tessmer MH, Stein RA, Feix JB, Mchaourab HS, Meiler J. Rapid simulation of unprocessed DEER decay data for protein fold prediction. Biophys J. 2019;2: 366–375. doi: 10.1016/j.bpj.2019.12.011 31892409 PMC6976798

[40] Tesei G, Martins JM, Kunze MBA, Wang Y, Crehuet R, Lindorff-Larsen K. DEER-PREdict: Software for efficient calculation of spin-labeling EPR and NMR data from conformational ensembles. Plos Comput Biol. 2021;17: e1008551. doi: 10.1371/journal.pcbi.1008551 33481784 PMC7857587

[41] Polyhach Y, Bordignon E, Jeschke G. Rotamer libraries of spin labelled cysteines for protein studies. Phys Chem Chem Phys. 2010;13: 2356–2366. doi: 10.1039/c0cp01865a 21116569

[42] Qi Y, Lee J, Cheng X, Shen R, Islam SM, Roux B, et al. CHARMM-GUI DEER facilitator for spin-pair distance distribution calculations and preparation of restrained-ensemble molecular dynamics simulations. J Comput Chem. 2020; 415–420. doi: 10.1002/jcc.26032 31329318

[43] Reichel K, Stelzl LS, Köfinger J, Hummer G. Precision DEER distances from spin-label ensemble refinement. J Phys Chem Lett. 2018;9: 5748–5752. doi: 10.1021/acs.jpclett.8b02439 30212206

[44] Islam SM, Roux B. Simulating the distance distribution between spin-labels attached to proteins. J Phys Chem B. 2015;119: 3901–3911. doi: 10.1021/jp510745d 25645890 PMC4509421

[45] Hagelueken G, Abdullin D, Ward R, Schiemann O. mtsslSuite: In silico spin labelling, trilateration and distance-constrained rigid body docking in PyMOL. Mol Phys. 2013;111: 2757–2766. doi: 10.1080/00268976.2013.809804 24954955 PMC4056886

[46] Hagelueken G, Abdullin D, Schiemann O. mtsslSuite: Probing biomolecular conformation by spin-labeling studies. Methods Enzymol. 2015;563: 595–622. doi: 10.1016/bs.mie.2015.06.006 26478500

[47] Smith JA, Edwards SJ, Moth CW, Lybrand TP. TagDock: an efficient rigid body docking algorithm for oligomeric protein complex model construction and experiment planning. Biochemistry. 2013;52: 5577–84. doi: 10.1021/bi400158k 23875708 PMC3804560

[48] Edwards SJ, Moth CW, Kim S, Brandon S, Zhou Z, Cobb CE, et al. Automated structure refinement for a protein heterodimer complex using limited EPR spectroscopic data and a rigid-body docking algorithm: A three-dimensional model for an ankyrin-CDB3 complex. J Phys Chem B. 2014;118: 4717–4726. doi: 10.1021/jp4099705 24758720 PMC4018176

[49] Jeschke G, Esteban-Hofer L. Integrative ensemble modeling of proteins and their complexes with distance distribution restraints. Methods Enzymol. 2022;666: 145–169. doi: 10.1016/bs.mie.2022.02.010 35465919

[50] Hatmal MM, Li Y, Hegde BG, Hegde PB, Jao CC, Langen R, et al. Computer modeling of nitroxide spin labels on proteins. Biopolymers. 2012;97: 35–44. doi: 10.1002/bip.21699 21792846 PMC3422567

[51] Michaud-Agrawal N, Denning EJ, Woolf TB, Beckstein O. MDAnalysis: a toolkit for the analysis of molecular dynamics simulations. J Comput Chem. 2011;32: 2319–27. doi: 10.1002/jcc.21787 21500218 PMC3144279

[52] Chaudhury S, Lyskov S, Gray JJ. PyRosetta: a script-based interface for implementing molecular modeling algorithms using Rosetta. Bioinformatics. 2010;26: 689–691. doi: 10.1093/bioinformatics/btq007 20061306 PMC2828115

[53] Schwieters CD, Kuszewski JJ, Clore GM. Using Xplor–NIH for NMR molecular structure determination. Prog Nucl Magn Reson Spectrosc. 2006;48: 47–62. doi: 10.1016/j.pnmrs.2005.10.001

[54] Schwieters CD, Kuszewski JJ, Tjandra N, Clore GM. The Xplor-NIH NMR molecular structure determination package. J Magn Reson. 2003;160: 65–73. doi: 10.1016/s1090-7807(02)00014-9 12565051

[55] Battiste JL, Wagner G. Utilization of site-directed sspin labeling and high-resolution heteronuclear nuclear magnetic resonance for global fold determination of large proteins with limited nuclear overhauser effect data. Biochemistry. 2000;39: 5355–5365. doi: 10.1021/bi000060h 10820006

[56] Tang C, Schwieters CD, Clore GM. Open-to-closed transition in apo maltose-binding protein observed by paramagnetic NMR. Nature. 2007;449: 1078–1082. doi: 10.1038/nature06232 17960247

[57] Judd M, Abdelkader EH, Qi M, Harmer JR, Huber T, Godt A, et al. Short-range ENDOR distance measurements between Gd (iii) and trifluoromethyl labels in proteins. Phys Chem Chem Phys. 2022;24: 25214–25226. doi: 10.1039/d2cp02889a 36222074

[58] Valentin MD, Albertini M, Farra MGD, Zurlo E, Orian L, Polimeno A, et al. Light-induced porphyrin-based spectroscopic ruler for nanometer distance measurements. Chem Eur J. 2016;22: 17204–17214. doi: 10.1002/chem.201603666 27868323

[59] Tessmer M, Canarie ER, Stoll S. Comparative evaluation of spin label modeling methods for protein structural studies. Biophys J. 2022. doi: 10.1016/j.bpj.2022.08.002 35957530 PMC9515001

[60] Berman HM, Westbrook J, Feng Z, Gilliland G, Bhat TN, Weissig H, et al. The Protein Data Bank. Nucleic Acids Res. 2000;28: 235–42. doi: 10.1093/nar/28.1.235 10592235 PMC102472

[61] Widder P, Schuck J, Summerer D, Drescher M. Combining site-directed spin labeling in vivo and in-cell EPR distance determination. Phys Chem Chem Phys. 2020;22: 4875–4879. doi: 10.1039/c9cp05584c 32072999

[62] Kugele A, Silkenath B, Langer J, Wittmann V, Drescher M. Protein spin labeling with a photocaged nitroxide using diels–alder chemistry. Chembiochem. 2019;20: 2479–2484. doi: 10.1002/cbic.201900318 31090999 PMC6790680

[63] Braun T, Drescher M, Summerer D. Expanding the genetic code for site-directed spin-labeling. Int J Mol Sci. 2019;20: 373. doi: 10.3390/ijms20020373 30654584 PMC6359334

[64] Warshaviak DT, Khramtsov VV, Cascio D, Altenbach C, Hubbell WL. Structure and dynamics of an imidazoline nitroxide side chain with strongly hindered internal motion in proteins. J Magn Reson. 2013;232: 53–61. doi: 10.1016/j.jmr.2013.04.013 23694751 PMC3758229

[65] Balo AR, Feyrer H, Ernst OP. Toward precise interpretation of DEER-based distance distributions: Insights from structural characterization of V1 spin-labeled side chains. Biochemistry. 2016;55: 5256–5263. doi: 10.1021/acs.biochem.6b00608 27532325

[66] Tormyshev VM, Chubarov AS, Krumkacheva OA, Trukhin DV, Rogozhnikova OYu, Spitsyna AS, et al. Methanethiosulfonate derivative of OX063 trityl: A promising and efficient reagent for site-directed spin labeling of Proteins. Chem Eur J. 2020;26: 2705–2712. doi: 10.1002/chem.201904587 31851392

[67] Jagtap AP, Krstic I, Kunjir NC, Hänsel R, Prisner TF, Sigurdsson STh. Sterically shielded spin labels for in-cell EPR spectroscopy: Analysis of stability in reducing environment. Free Radical Res. 2014;49: 78–85. doi: 10.3109/10715762.2014.979409 25348344

[68] Mchaourab HS, Kálai T, Hideg K, Hubbell WL. Motion of spin-labeled side chains in T4 lysozyme: Effect of side chain structure. Biochemistry. 1999;38: 2947–2955. doi: 10.1021/bi9826310 10074347

[69] Yang Y, Yang F, Gong Y-J, Bahrenberg T, Feintuch A, Su X-C, et al. High sensitivity In-cell EPR distance measurements on proteins using an optimized Gd(III) spin label. J Phys Chem Lett. 2018;9: 6119–6123. doi: 10.1021/acs.jpclett.8b02663 30277780

[70] Kugele A, Braun TS, Widder P, Williams L, Schmidt MJ, Summerer D, et al. Site-directed spin labelling of proteins by Suzuki–Miyaura coupling via a genetically encoded aryliodide amino acid. Chem Commun. 2019;55: 1923–1926. doi: 10.1039/c8cc09325c 30680379

[71] Pracht P, Bohle F, Grimme S. Automated exploration of the low-energy chemical space with fast quantum chemical methods. Phys Chem Chem Phys. 2020;22: 7169–7192. doi: 10.1039/c9cp06869d 32073075

[72] Holm L, Sander C. Database algorithm for generating protein backbone and side-chain co-ordinates from a Cα trace Application to model building and detection of co-ordinate errors. J Mol Biol. 1991;218: 183–194. doi: 10.1016/0022-2836(91)90883-8 2002501

[73] Shapovalov MV, Dunbrack RL. A smoothed backbone-dependent rotamer library for proteins derived from adaptive kernel density estimates and regressions. Structure. 2011;19: 844–58. doi: 10.1016/j.str.2011.03.019 21645855 PMC3118414

[74] Frazier AA, Wisner MA, Malmberg NJ, Victor KG, Fanucci GE, Nalefski EA, et al. Membrane orientation and position of the C2 domain from cPLA2 by site-directed spin labeling. Biochemistry. 2002;41: 6282–92. doi: 10.1021/bi0160821 12009889

[75] Yu L, Fang W, He Y, Cai W, Wei W, Tian C. Secondary structure and transmembrane topology analysis of the N-terminal domain of the inner membrane protein EccE1 from M. smegmatis using site-directed spin labeling EPR. Biochim Biophys Acta, Biomembranes. 2021;1863: 183515. doi: 10.1016/j.bbamem.2020.183515 33245893

[76] Francis DJ, Hubbell WL, Klug CS. Probing protein secondary structure using EPR: Investigating a dynamic region of visual arrestin. Appl Magn Reson. 2012;43: 405–419. doi: 10.1007/s00723-012-0369-y 25419051 PMC4240029

[77] Yang L, Adam C, Nichol GS, Cockroft SL. How much do van der Waals dispersion forces contribute to molecular recognition in solution? Nat Chem. 2013;5: 1006–1010. doi: 10.1038/nchem.1779 24256863

[78] Spicher S, Grimme S. Robust atomistic modeling of materials, organometallic, and biochemical systems. Angew Chem Int Ed. 2020; 15665–15673. doi: 10.1002/anie.202004239 32343883 PMC7267649

[79] Spicher S, Abdullin D, Grimme S, Schiemann O. Modeling of spin–spin distance distributions for nitroxide labeled biomacromolecules. Phys Chem Chem Phys. 2020;22: 24282–24290. doi: 10.1039/d0cp04920d 33107523

[80] Yao K, Herr JE, Toth DW, Mckintyre R, Parkhill J. The TensorMol-0.1 model chemistry: a neural network augmented with long-range physics. Chem Sci. 2018;9: 2261–2269. doi: 10.1039/c7sc04934j 29719699 PMC5897848

[81] Gao X, Ramezanghorbani F, Isayev O, Smith JS, Roitberg AE. TorchANI: A free and open source PyTorch-based deep learning implementation of the ANI neural network potentials. J Chem Inf Model. 2020;60: 3408–3415. doi: 10.1021/acs.jcim.0c00451 32568524

[82] Anderson DM, Feix JB, Monroe AL, Peterson FC, Volkman BF, Haas AL, et al. Identification of the major ubiquitin-binding domain of the Pseudomonas aeruginosa ExoU A2 phospholipase. J Biol Chem. 2013;288: 26741–26752. doi: 10.1074/jbc.M113.478529 23908356 PMC3772220

[83] Tessmer MH, Anderson DM, Pickrum AM, Riegert MO, Moretti R, Meiler J, et al. Identification of a ubiquitin-binding interface using Rosetta and DEER. Proc Nat Acad Sci USA. 2018;115: 525–530. doi: 10.1073/pnas.1716861115 29295930 PMC5776994

[84] Anderson DM, Schmalzer KM, Sato H, Casey M, Terhune SS, Haas AL, et al. Ubiquitin and ubiquitin-modified proteins activate the Pseudomonas aeruginosa T3SS cytotoxin, ExoU. Mol Microbiol. 2011;82: 1454–67. doi: 10.1111/j.1365-2958.2011.07904.x 22040088 PMC3237844

[85] Georgieva ER, Roy AS, Grigoryants VM, Borbat PP, Earle KA, Scholes CP, et al. Effect of freezing conditions on distances and their distributions derived from Double Electron Electron Resonance (DEER): a study of doubly-spin-labeled T4 lysozyme. J Magn Reson. 2012;216: 69–77. doi: 10.1016/j.jmr.2012.01.004 22341208 PMC3323113

