# Supplementary material for: chiLife: An open-source Python package for in silico spin labeling and integrative protein modeling
Source: PLoS Comput Biol. 2023 Mar 31;19(3):e1010834. doi: 10.1371/journal.pcbi.1010834 (PMC10096462; doi:10.1371/journal.pcbi.1010834)
Supplement: S1 Text — (PDF) [file pcbi.1010834.s001.pdf]

Supporting Information

chiLife: An open-source Python package for *in silico* spin labeling and integrative protein modeling

Maxx H. Tessmer, Stefan Stoll

Department of Chemistry, University of Washington, Seattle, WA 98103, United States

Listing S1: Solvent-accessible SDSL screen for maximal distance contrast

```
from itertools import combinations
import numpy as np
from scipy.stats import wasserstein_distance
import chilife as xl

r = np.linspace(0, 100, 256)

unbound = xl.fetch('1omp')
bound = xl.fetch('1anf')

# Get residues that are solvent accessible in both conformations
unbound_SASR = xl.get_sas_res(unbound, 50)
bound_SASR = xl.get_sas_res(bound, 50)
SASR = unbound_SASR & bound_SASR

Ps, SL_pairs = [], []
for protein in (unbound, bound):
    # Create SpinLabels at shared solvent accessible sites
    SLs = {site: xl.SpinLabel('R1M', site, protein, chain=chain)
                for site, chain in SASR}

    # Calculate distance distributions between all pairs
    pairs = list(combinations(SLs.values(), r=2))
    SL_pairs.append(pairs)
    Ps.append(np.array([xl.distance_distribution(SL1, SL2, r) for SL1, SL2 in pairs]))

# Sort site pairs by the earth movers distance between the bound and unbound state
EMDs = [wasserstein_distance(r, r, P_unbound, P_bound) for P_unbound, P_bound in zip(*Ps)]
args = np.argsort(EMDs)[::-1]

unbound_SL1, unbound_SL2 = SL_pairs[0][arg]
bound_SL1, bound_SL2 = SL_pairs[1][arg]

unbound_P = Ps[0][args[0]]
bound_P = Ps[1][args[0]]
```

Listing S2: Creation of new chiLife rotamer libraries

```
weights = np.loadtxt('R3A/weights.txt')
xl.create_library('R3A', 'R3A/R3A_Ensemble.pdb',
                  dihedral_atoms=[['N', 'CA', 'CB', 'SG'],      # Name of the atoms defining chi1 dihedral
                                  ['CA', 'CB', 'SG', 'CD'],     # chi2 dihedral
                                  ['CB', 'SG', 'CD', 'C3'],     # chi3 dihedral
                                  ['SG', 'CD', 'C3', 'C4']],    # chi4 dihedral
                  spin_atoms=['N1', 'O1'],                      # Define spin atoms
                  weights=weights)

weights = np.loadtxt('NBA/weights.txt')
xl.create_library('NBA', 'NBA/NBA_Ensemble.pdb',
                  site=2,                                       # Libraries can be created from any site
                  dihedral_atoms=[['N', 'CA', 'C01', 'C07'],
                                  ['CA', 'C01', 'C07', 'C08'],
                                  ['C03', 'C04', 'C09', 'C13'],
                                  ['C10', 'C11', 'N01', 'C14'],
                                  ['N01', 'C14', 'C15', 'C16']],
                  spin_atoms={'N02': 0.5, 'O01': 0.5},          # Spin atoms can be given weights
                  weights=weights)

weights = np.loadtxt('GD3/weights.txt')
xl.create_library('GD3', 'GD3/GD3_Ensemble.pdb',
                  dihedral_atoms=[['N', 'CA', 'CB', 'SG'],
                                  ['CA', 'CB', 'SG', 'C18'],
                                  ['CB', 'SG', 'C18', 'C19'],
                                  ['C17', 'C16', 'C9', 'N1'],
                                  ['C16', 'C9', 'N1', 'C8']],
                   spin_atoms='Gd1',                            # Single spin atom doesn't need a list
                  weights=weights)


T4L = xl.fetch('2lzm')

# Create a spin label with explicit declaration
T109R3A = xl.SpinLabel('R3A', 109, T4L, rotlib='R3A_rotlib.npz')

# The extension does not need to be specified for the rotlib
T109NBA = xl.SpinLabel('NBA', 109, T4L, rotlib='NBA')

# rotlib doesn't need to be specified if has the same name as the resname and is in the working directors
T109GD3 = xl.SpinLabel('GD3', 109, T4L, sample=5000)

xl.save(T109R3A, T109NBA, T109GD3, T4L)
```

Listing S3: Local side chain repacking

```
mbp = xl.fetch('1omp')
SL1 = xl.SpinLabel('R1M', 124, mbp)
SL2 = xl.SpinLabel('R1M', 281, mbp)

traj, dE = xl.repack(mbp, SL1, SL2,
                     repetitions=5000, temp=298,
                     off_rotamer=True,
                     repack_radius=10)

SL1_repack = xl.SpinLabel.from_trajectory(traj, 124, burn_in=1000, spin_atoms=SL1.spin_atoms)
SL2_repack = xl.SpinLabel.from_trajectory(traj, 281, burn_in=1000, spin_atoms=SL2.spin_atoms)
```

Listing S4: Membrane docking

```
import numpy as np
from scipy.spatial.transform import Rotation
from scipy.optimize import minimize
import chiLife as xl

# Get protein and membrane depth data
CPLA_C2 = xl.fetch('1bci').select_atoms('protein')
sites, depths = np.loadtxt('Membrane_depths.txt')

# Create labels and extract centroid
label_list = [xl.SpinLabel('R1A', site, protein= CPLA_C2, sample=1000) for site in sites]
spin_centroids = [SL.spin_centroid for SL in label_list]

# Translate array of spin centroids to the origin for rotating
spin_at_ori = spin_centroids - np.mean(spin_centroids, axis=0)

# Create the objective function to fit Euler angles and membrane depth to the data
def objective(par):
    x, y, z, zdepth = par

    # Create a rotation matrix from the Euler angles
    R = Rotation.from_euler('xyz', [x, y, z])
    M = R.as_matrix()

    # Rotate and translate the spins
    spin_coords = spin_at_ori@M + np.array([0, 0, zdepth])

    # Compare the spin depth to the experimental depths and return the sum of squares error
    resid = spin_coords[:, 2] - depths
    return resid @ resid


fit = minimize(objective, x0=[0, 0, 0, 0], bounds=[[-180, 180], [-180, 180], [-180, 180], [-100, 100]])
```

Listing S5: Protein-protein docking with chiLife and PyRosetta

```
import sys, os, pickle, json
from pathlib import Path
from dataclasses import dataclass
from pyrosetta import *
import numpy as np
import chiLife as xl
from rosetta.core.scoring.methods import WholeStructureEnergy
from pyrosetta.rosetta.core.io.raw_data import ScoreMap
from pyrosetta.rosetta.protocols.geometry import center_of_mass
from pyrosetta.rosetta.protocols.docking import setup_foldtree, DockingProtocol, calc_interaction_energy

init()
pose = pose_from_pdb('complex.pdb')
native_pose = pose_from_pdb('complex_20145.pdb')
```

```python
to_centroid = SwitchResidueTypeSetMover('centroid')
to_full_atom = SwitchResidueTypeSetMover('fa_standard')

ub_offset = 629
exou_offset = 54

@dataclass
class ExpData:
    name: str
    site1: int
    site2: int
    label1: str
    label2: str
    r: np.ndarray
    P: np.ndarray

@EnergyMethod()
class RosettaDipolarDistance(WholeStructureEnergy):
    def __init__(self):
        WholeStructureEnergy.__init__(self, self.creator())

        # Load in experimental data
        with open('ExpData.pkl', 'rb') as f:
            self.data_dict = pickle.load(f)

        self.sites = []
        self.pose_sites = []
        self.labels = []
        for site1, site2 in self.data_dict:

            if site1 not in self.sites:
                self.sites.append(site1)
                self.pose_sites.append(site1 + ub_offset)
                self.labels.append(self.data_dict[(site1, site2)].label1)

            if site2 not in self.sites:
                self.sites.append(site2)
                self.pose_sites.append(site2 - exou_offset)
                self.labels.append(self.data_dict[(site1, site2)].label2)

        self.SLCache = {}
        self.iteration = 0
        self.last_score = 1e6

    def setup_for_scoring(self, pose, sf):
        # Create MDA Universe if it does not exist
        if not hasattr(self, 'mda_protein'):
            if not pose.is_fullatom():
                to_full_atom.apply(pose)
                self.mda_protein = xl.pose2mda(pose)
                to_centroid.apply(pose)
            else:
                self.mda_protein = xl.pose2mda(pose)

            self.SLCache = {site: xl.SpinLabel(label, site, self.mda_protein)
                            for label, site in zip(self.labels, self.pose_sites)}

        # Recalculate rotamer ensembles every 10 iterations
        if self.iteration % 10 == 0 and pose.is_fullatom() and self.iteration != 0:
            # Update the universe with rosetta coordinates
            self.mda_protein.atoms.positions = np.array([res.xyz(atom)
                                                         for res in pose.residues
                                                         for atom in range(1, res.natoms() + 1)
                                                         if res.atom_type(atom).element().strip() != 'X'])

            # Recalculate spin labels
            self.SLCache = {site: xl.SpinLabel(label, site, self.mda_protein)
                            for label, site in zip(self.labels, self.pose_sites)}
```

```python
        else:
            # Just move the spin label ensemble with the protein
            for site in self.pose_sites:
                backbone =  np.array([pose.residue(site).xyz('N'),
                                      pose.residue(site).xyz('CA'),
                                      pose.residue(site).xyz('C')])
                self.SLCache[site].to_site(backbone)

        self.iteration += 1

    def finalize_total_energy(self, pose, efunc, emap):

        score = 0
        for r1, r2 in self.data_dict:

            # Calculate preidcted P(r)s
            r= self.data_dict[(r1, r2)].r
            P = self.data_dict[(r1, r2)].P
            SL1, SL2 = self.SLCache[r1 + ub_offset], self.SLCache[r2 - exou_offset]
            dd = xl.distance_distribution(SL1, SL2, r).clip(0)

            # If the distance distribution is outside the provided domain (r)
            if np.any(np.isnan(dd)):

                CA_begin = pose.chain_begin(1)
                CB_begin = pose.chain_begin(2)
                CA_end = pose.chain_end(1)
                CB_end = pose.chain_end(2)

                diff = np.array(center_of_mass(pose, CA_begin, CA_end)) –
                       np.array(center_of_mass(pose, CB_begin, CB_end))

                cen_dist = np.sqrt(diff @ diff)

                # don’t alter score when calculating ddg
                if cen_dist > 1000:
                    score = self.last_score
                    break

                # Otherwise penalize the difference between the mode distance to the cbeta distance
                else:
                    diff = np.array(pose.residue(r1).xyz('CA')) - np.array(pose.residue(r2).xyz('CA'))
                    CAdist = np.sqrt(diff @ diff)
                    score += np.abs(CAdist - r[np.argmax(P)])

            else:
                # Normalize and calculate overlap score
                P /= P.sum()
                dd /= dd.sum()

                score += -np.sum(np.minimum(P.clip(0), dd.clip(0)))

        self.last_score = score
        emap.set(self.scoreType, score)

# Create score functio and add dipolar distance term
RDD = RosettaDipolarDistance.scoreType
weight = 5
sfhr = create_score_function('docking')
sflr = create_score_function('interchain_cen')
sfhr.set_weight(RDD, weight)
sflr.set_weight(RDD, weight)

# Setup docking protocol
docking_protocol = DockingProtocol()
docking_protocol.set_highres_scorefxn(sfhr)
docking_protocol.set_lowres_scorefxn(sflr)
docking_protocol.set_partners('A_B')
```

```python
# Setup for global docking
prtrbr = docking_protocol.perturber()
prtrbr.set_randomize1(True)
prtrbr.set_randomize2(True)
prtrbr.set_spin(True)

setup_foldtree(pose, "A_B", Vector1([1]))

nsuccess = 0
while nsuccess < 100:

    # Check to see if the decoy exists (checkpointing)
    newname = f'decoys/complex_{int(sys.argv[1]) * 100 + nsuccess}.pdb'
    if Path(newname).exists():
        print(f'{newname} alread exists skipping...')
        nsuccess += 1
        continue

    # Run protocol on a copy of the input pose
    pose2 = pose.clone()
    docking_protocol.apply(pose2)

    # Skip if filtered out by docking protocol
    if not pose2.is_fullatom():
        print('Pose is not FA. Skipping output')
        continue

    # compute additional score terms
    total = sfhr(pose2)
    scores = dict(list(ScoreMap.get_energies_map_from_scored_pose(pose2).items()))
    I_sc = calc_interaction_energy(pose2, sfhr, Vector1([1]))
    RMSD1 = pyrosetta.rosetta.core.scoring.CA_rmsd(native_pose, pose2)
    scores.update({'I_sc': I_sc, 'rmsd': RMSD1, 'name': newname})

    # Save to score file and pdb
    with open(f'scores_{sys.argv[1]}.sc', 'a') as f:
        json.dump(scores, f)
        f.write('\n')

    pose.dump_pdb(newname)
    nsuccess += 1
```
