# Supplementary material for: chiLife: An open-source Python package for in silico spin labeling and integrative protein modeling
Source: PLoS Comput Biol. 2023 Mar 31;19(3):e1010834. doi: 10.1371/journal.pcbi.1010834 (PMC10096462; doi:10.1371/journal.pcbi.1010834)
Supplement: Attachment — Submitted filename: chiLife_rev1_reviewer_response.pdf [file pcbi.1010834.s002.pdf]

RESPONSE TO REVIEWERS

We thank the reviewers for their valuable feedback. Based on reviewer input we have made a few modifications to the manuscript that we believe add to the overall value of this work. We have compiled a point-by-point response to the reviewer's comments and appended it below.

Reviewer 1

In their article "chiLife: An open-source Python package for in silico spin labelling and integrative protein modelling", Tessmer and Stoll present a very nice piece of software that can be easily integrated into other Python code, allowing programmers to easily attach ensembles of spin labels to protein models and perform a number of calculations with such models.

The package is well designed, open source and the description in the paper is very good. The authors have included a number of examples and code listings which will make it easy for users to learn how to use the library.

This library will be an important addition to the field of spin-labelling EPR and I think, due to the way it is constructed, it will also be very useful in other fields such as FRET.

It would be great if the package could also work with nucleic acids. I guess, such a feature is already on the list for an upcoming version.

This is indeed a planned feature for a future release.

We have opened a new issue on the chiLife GitHub repository to track the progress of this feature.

I recommend publication of this paper.

Some minor points:

1.It is not entirely clear to me how the fetch method deals with models that have multiple copies in the ASU - is it possible to fetch a specific chain of the model, e.g. using xl.fetch('1omp_A') or something similar? Will the user be warned that there are multiple chains?

This is an excellent point. chiLife does *not* warn the user about multiple chains and assumes the user knows the pdb file they are fetching and does not offer chain-specific fetching; however, the fetch function does return an MDAnalysis Universe object which has a rich atom selection feature that can in turn be used to select specific chains or other substructures. Users can define their own atom selection from the fetched protein and chiLife will respect that selection for all calculations including clash evaluations, solvent accessibility, and saving.

We have now added a brief discussion stating explicitly that fetch returns an MDAnalysis Universe object and describing how chiLife interacts with these objects so that users can accurately reconstruct the protein environment they are trying to model in.

2.PDB files sometimes contain ligands and solvent molecules that can interfere with calculations but are difficult to detect automatically. Sometimes it makes sense to include them, sometimes not. It would be great if the program could output a stripped-down version of the input .pdb file, containing all the atoms used in the calculations. Is such a method already included?

We agree with the reviewer that solvent and ligands can be important factors that users may wish to include or exclude when modeling spin labels. With the exception of water molecules, chiLife will consider the effect of ligands and other solvents when modeling side chains so long as they are included in the MDanalysis Universe or AtomGroup provided by the user. As described above, MDAnalysis can be used to remove undesired ligands or solvents from the system and chiLife will respect user-defined atom selections.

As for the previous comment, here we choose not to replicate in chiLife functionality that is already available, well implemented, and widely used in MDAnalysis. This leverages the mature Python ecosystem for biostructural modeling.

We have now included a better description of atom selection and how chiLife interacts with it, as well as an additional comment about the implicit exclusion of water when modeling.

3.Are bipedal spin labels such as Rx or the Cu-NTA label supported?

While bifunctional spin label modeling is not yet formally supported, chiLife can already be used to model bifunctional spin labels. The code is already available via the chiLife repository on GitHub.

While we are excited about modeling bifunctional labels, we consider inclusion of this beyond the scope of this manuscript. The main reason is that our approach uses a novel method of attachment for bifunctional labels that has not yet been fully validated experimentally. Accordingly, we did not modify the manuscript to include any discussion of bifunctional labels.

Reviewer 2

In the article 'chiLife: An open-source Python package for in silico spin labeling and integrative protein modeling' the authors publish an truly useful follow-up article to their landmark paper 'Comparative evaluation of spin-label modeling methods for protein structural studies'. In the article the authors introduce and further provide detailed insights to chiLife package. The chiLife package has the capability to correctly model paramagnetic labels on biomolecuels that eventually lead information such as their distance between them and solvent accessibility. However the most striking ability is that it is written fully in python that makes it freely accessible and foremost easily integrated into other programs such as NIH-Xplor, pyRosetta and others. This is a big step forward to programs such as DeerRosetta and MMM.

The authors lead the reader through the article with numerous examples that become widely accessible to the none-EPR expert. Most important the reader can subsequently integrate chiLife in his/her own research. I deeply enjoyed and appreciated the overlap between the experimental and simulated distances presented in figure 1 and 7, that show the correct modeling approaches used in the publication. I appreciate the ease on how to add costume labels to chiLife that will become more useful in the timeline of bio-molecular EPR. Once labels are added to the biomolecule the local environment can be repacked via customized scoring function. Now while repacking has been applied to many systems before, here the authors introduce a 'scoring function', which gives the user the ultimate freedom to apply to wide range of systems that stretch further than the normal protein system. However I would like to have more information on how to apply those scoring function.

We appreciate reviewer 2’s recognition of the utility and potential of applying custom score functions to spin-label modeling. After reviewing the relevant section, we agree that the description of adding custom score functions can be improved.

We have now moved Listing S5 into figure 7 and adjusted the text accordingly. We hope this provides readers with a clear illustration of how they can utilize custom score functions in chiLife.

Lastly the authors treat an under estimated strength of EPR, which is the membrane docking function and solvent accessibility. Since the age of Hubell and side directed spin labeling researchers have used EPR to get information on localized environment that changes upon applying molecular stimuli. Specifically a study by Ralf Langen treated the membrane remodeling ability introduced by the bar domain. Here, we have a tool that can correctly interpret such data and see domain reorientation that introduce specific biological functions.

Overall, I want to recommend the article for publication. One slight addition would be a specific

outline on how to install the software, which is in detail provided on the chiLife website with all other tutorials (see 'Availability').

Accessibility and ease of installation are important to us.

We have now made chiLife available on the Python package index (PyPi) to facilitate easy installations and added a brief description of the installation process in the “Availability” section.
